# A classification system for youth outpatient behavioral health services billed to medicaid

**DOI:** 10.3389/frhs.2024.1298592

**Published:** 2024-02-05

**Authors:** Gabriela M. Rodríguez, Casey A. Pederson, Dainelys Garcia, Katherine Schwartz, Steven A. Brown, Matthew C. Aalsma

**Affiliations:** ^1^ Department of Psychiatry, Indiana University School of Medicine, Indianapolis, IN, United States; ^2^ Department of Pediatrics, University of Miami Miller School of Medicine, Miami, FL, United States; ^3^ Department of Pediatrics, Indiana University School of Medicine, Indianapolis, IN, United States; ^4^ Department of Biostatistics and Health Data Science, Indiana University School of Medicine, Indianapolis, IN, United States

**Keywords:** behavioral health services, administrative data, youth mental health, Medicaid, procedure codes

## Abstract

Rates of youth behavioral health concerns have been steadily rising. Administrative data can be used to study behavioral health service utilization among youth, but current methods that rely on identifying an associated behavioral health diagnosis or provider specialty are limited. We reviewed all procedure codes billed to Medicaid for youth in one U.S. county over a 10-year period. We identified 158 outpatient behavioral health procedure codes and classified them according to service type. This classification system can be used by health services researchers to better characterize youth behavioral health service utilization.

## Introduction

Outpatient behavioral healthcare utilization patterns are understudied, especially among children and adolescents (1). Behavioral healthcare is inclusive of interventions designed to address mental (e.g., depression, conduct) and/or behavioral (e.g., substance use) health concerns. Given rising rates of these concerns among youth, it is critical that we understand how these problems are being treated. However, types of behavioral healthcare provided in the community can vary widely (e.g., skills training vs. therapy). Thus, characterizing utilization patterns requires a standard way to describe the types of behavioral health services furnished to youth in a community setting. Previous research in the U.S. has used administrative data, such as the data routinely collected by healthcare systems to bill for services provided, to characterize behavioral health service utilization with various service classification methods. While administrative data is a powerful tool for identifying behavioral health service utilization patterns for children and adolescents, there is a dearth of guidance on how best to classify the services used by youth.

Previous studies using administrative data to describe service utilization have relied on a combination of diagnosis (e.g., F90.9, Attention-Deficit Hyperactivity Disorder) and lists of procedure codes (e.g., 90834, Psychotherapy, 45 min with patient; 90847, Family psychotherapy with patient present, 50 min) selected *a priori* by the research team to characterize behavioral health services (1–3). Other studies using administrative data have classified services as behavioral health-related by identifying those billed by a mental health specialist or associated with specific psychiatric diagnostic codes. In their study of healthcare utilization for children with mental health conditions enrolled in Medicaid across 11 states, Doupnik and colleagues (4) used provider taxonomy codes (e.g., psychiatrist, psychologist), such that if a billing claim included a mental health specialist and/or was associated with an ICD-10-CM code for a mental health diagnosis, it was classified as a mental health ambulatory office visit. In another study, a combination of provider taxonomy codes, procedure codes, place of service codes, and psychiatric diagnosis codes were used to identify ambulatory mental health visits before and after a suicidal crisis among children and adolescents (5).

Though there is some overlap in the methods described to identify behavioral health services within administrative healthcare datasets, there are some challenges that limit their utility. One limitation is the absence of individual provider taxonomy codes in Medicaid billing data for some states. For example, in a study aiming to use Medicaid claims data for 17 states to investigate reimbursement differences between psychiatrists and primary care physicians, five states had to be excluded from the analyses because more than 75% of claims were missing a provider taxonomy code (6). Another limitation relates to changes in procedure codes over time. For example, several procedure codes for individual psychotherapy were discontinued and replaced with new codes in 2013 (7). Using only active procedure codes would thus lead to inadvertently excluding some behavioral health services when examining historical billing claims data. Differences in how providers document diagnoses for billing purposes (e.g., documenting only one out of multiple active diagnoses, hesitation to document stigmatized diagnoses) can also limit capacity to capture all behavioral health visits.

Finally, there are important differences among types of behavioral health services. Grouping services together into broadly defined “psychosocial” or “behavioral health” visit counts limits characterization of the treatment received. Services that may be related to behavioral health beyond psychotherapy, such as assessment, psychological testing, and case management, could be missed using existing methods. Our current goal is to address these limitations by reviewing and classifying standardized procedure codes according to behavioral health service type, without relying on diagnosis or detailed provider taxonomy codes. We reviewed all codes billed to Medicaid on an outpatient basis for youth patients during a 10-year period within one large, Midwest county in the U.S. The primary purpose of this work is to share our comprehensive coding scheme to help health services researchers better leverage administrative data to study and describe youth outpatient behavioral health service utilization.

## Methods

We undertook this work as part of a larger study aiming to characterize behavioral health service utilization among youth involved in the juvenile legal system during the year following arrest. One long-term aim of the larger study is to inform how to best leverage psychosocial services to deter youth from requiring higher levels of care. Thus, the service types of interest for the current study included outpatient psychosocial services and did not include more intensive services, such as inpatient and residential, or medication-related services. Accordingly, for this study, behavioral health services were defined as psychosocial interventions designed to address mental (e.g., depression, conduct) and/or behavioral (e.g., substance use) disorders as provided by a mental healthcare provider.

Data were collected as part of a retrospective study of Medicaid billing claims data for youth aged 8–17 years in one Midwest county (population ∼1 million) during 2007–2017. The claims dataset reviewed by researchers for this study was accessed through a data use agreement between the state's Family and Social Services Administration and university researchers. The raw dataset included data related to all approved Medicaid claims during the study period, regardless of Medicaid coverage plan. For each approved claim, data included variables describing the care recipient (e.g., MRN, demographics) and service received (e.g., location of service, provider categorization, procedure codes, and diagnoses associated with each billed service). For this study, all patient-related data were removed from service descriptors before the data were coded.

Previous studies using this type of administrative data have used lists of procedure codes selected *a priori* to identify behavioral health services and could, therefore, fail to identify relevant services with procedure codes used in different settings or by different specialties. To address this limitation, we elected to start from all procedure codes in the database. To narrow the procedure codes to be reviewed – independent of related behavioral health diagnoses – we first attempted to review all codes billed from the state's most common source of behavioral healthcare (i.e., community mental health centers) using the place of service code to narrow the dataset. We identified 152 unique procedure codes billed with “community mental health center” as the place of service. However, we later learned the state Medicaid program enrolls several specialties under one provider type, “Behavioral Health Provider,” which includes community mental health centers as well as several other types of behavioral healthcare providers (e.g., outpatient mental health clinics, licensed psychologists, licensed clinical social workers, licensed mental health counselors, etc.). To capture as many outpatient behavioral health services as possible, we expanded the scope of our investigation beyond community mental health centers and reviewed all procedure codes (*n* = 1,469) billed by a “Behavioral Health Provider” for youth in our sample.

The Healthcare Common Procedure Coding System (HCPCS), produced by the U.S. Centers for Medicare and Medicaid Services, is a collection of standardized codes that represent medical services, procedures, supplies, and products, and is divided into two subsystems, or “levels.” Level I is comprised of Current Procedural Terminology (CPT, also known as “procedure codes”) Category I, a system of 5-digit numeric codes maintained by the American Medical Association (AMA) and used to identify medical services and procedures delivered by health care professionals. Level II codes consist of a letter followed by four numeric digits and identify products, supplies, and services not included in CPT codes (8, 9). Additionally, Category II codes are supplemental tracking codes used for quality and performance measurement that can be billed individually or along with a CPT Category I procedure code (and are distinct from Level II codes). We used the CPT, Level I and the HCPCS, Level II data files published by the AMA to obtain descriptions for 1,211 procedure codes. Additional procedure code descriptions were obtained from code tables published on the state Medicaid website (*n* = 11) and on the federal Medicaid website (*n* = 10). We conducted online searches to obtain descriptions for the remaining service codes (*n* = 237), some of which had been discontinued (e.g., 90804-12) in 2013 and 2018, and thus, did not appear in the current AMA files.

Next, we developed definitions for each of the service types of interest (see Table 1), which were then applied independently by coders (authors GR and CP) to classify each procedure code. We classified all procedure codes associated with outpatient psychosocial services, including assessment, psychological testing, therapy, group therapy, crisis therapy, and case management/skills training. The two initial coders are licensed clinical psychologists who provide behavioral health services to youth within the county of interest. Coders met periodically during the classification process to discuss and resolve coding discrepancies. A third licensed clinical psychologist (author DG) who provides behavioral health services in a different state reviewed the procedure code descriptions and classifications. All three licensed psychologists then met to discuss and resolve any remaining coding discrepancies.

**Table 1:** Service classification definitions and procedure codes.

Classification	Codes	Definition
Assessment	90791, 90792, 90801, 90802, 96103, 96105, 96110, 96111, 96127, 96150, 96151, 96156, 99408, 99409, G0396, G0397, G0442, G0444, G0469, H0001, H0002, H0031, H0049, H1011	Services designed to assess the current functioning of a patient that is a part of routine treatment services. This does not include more formal testing such as developmental or neuropsychological testing. Both in person and telemedicine appointments are included here.
Case management/skills training	90882, 97535, 97537, G9012, H0006, H0023, H0030, H0038, H2014, H2015, H2016, H2017, H2018, H2021, T1012, T1016, T1017, T1018, T2022, T2023	Individual or family case management or skills training with a treatment provider in an outpatient setting, wherein the patient has the ability to go home or resume regular activities following the appointment.
Category II	1000F, 1036F, 1040F, 1111F, 1124F, 1220F, 1494F, 3016F, 3085F, 3092F, 4000F, 4004F	CPT Category II Codes are supplemental tracking codes used for performance measurement and data collection related to quality and performance measurement, including Healthcare Effectiveness Data and Information Set (HEDIS®).
Crisis therapy	90839, 90840, H0007, H2011, S9484, S9485	Services that are time-limited with a specific psychotherapeutic approach to immediately stabilize those in crisis.
Outpatient group therapy	90849, 90853, 90857, 96153, 96164, 96165, H0005	Group therapy or intervention services occurring on an outpatient basis. Outpatient therapy includes therapy conducted in a community mental health center, wherein the patient is able to go home afterwards, or home-based therapy, wherein they provider may provide services in the clients home but leave afterwards. This also includes outpatient therapy occurring in person or via telemedicine.
Outpatient therapy	90785, 90804, 90805, 90806, 90807, 90808, 90809, 90810, 90811, 90812, 90814, 90815, 90816, 90817, 90818, 90819, 90821, 90832, 90833, 90834, 90836, 90837, 90838, 90843, 90845, 90846, 90847, 90855, 90875, 90876, 90880, 90934, 90984, 96152, 96154, 96155, 96158, 96159, 96167, 96168, 96170, 96171, 97532, 99406, 99407, 99510, 90832 SC, 90833 SC, 90834 SC, 90836 SC, 90837 SC, 90838 SC, 99401 HK, 99407 U6, G0176, G0436, G0443, H0004, H0015, H0050, H2019, H2020, H2035, T1006	Individual or family therapy or intervention services occurring on an outpatient basis. Outpatient therapy includes therapy conducted in a community mental health center, wherein the patient is able to go home afterwards, or home-based therapy, wherein they provider may provide services in the clients home but leave afterwards. This also includes outpatient therapy occurring in person or via telemedicine.
Psychological testing	90887, 96101, 96102, 96116, 96118, 96119, 96120, 96138, 96139, 96146	Services designed to assess the current functioning of a patient that is not a part of routine treatment services. This includes more formal testing such as developmental or neuropsychological testing. Both in person and telemedicine appointments are included here.
Unspecified behavioral health service	90854, 90887, 90899, G0155, G0176, G0177, G0409, G0470, H0036, H0037, H0039, H0040, H0046, H2034, H2036	Behavioral health service without enough information to classify further.

## Results

Of the 1,469 procedure codes billed by a “Behavioral Health Provider”, 158 were identified as outpatient behavioral health services. See Table 1 for our categorization of these 158 codes. We classified 24 codes as assessment, 20 as case management/skills training, 6 as crisis therapy, 7 as outpatient group therapy, 64 as outpatient therapy, and 10 as psychological testing. We also identified 12 Category II codes. Another 15 codes were classified as “unspecified behavioral health service,” as the description provided enough information to determine the service was related to behavioral health, but not enough information to classify further (e.g., H0046, Mental Health Services, Not Otherwise Specified).

Additional procedure codes that were identified as behavioral health services but were not classified further as they were beyond the scope of this manuscript included: administrative codes, psychiatry/medication management services (both outpatient and inpatient), drug screening or testing services, inpatient behavioral health services, residential services, day treatment or partial hospitalization services, and general medical services or procedures. We did not find descriptions for 116 procedure codes either in the AMA files or through online searches, so these codes were not classified. We suspect several of these codes represent data entry errors (e.g., fewer than 5 digits entered, 5 letters entered with no numbers, etc.).

## Discussion

Research examining youth behavioral health service utilization is limited, and methods for classifying services according to type of behavioral health service provided when using administrative data are needed. We reviewed and classified all outpatient procedure codes billed to Medicaid for youth in one county in the U.S. between 2007 and 2017. We identified 158 behavioral health service codes and classified them according to service type (assessment, case management/skills training, Category II, crisis therapy, outpatient group therapy, outpatient therapy, psychological testing, and unspecified behavioral health service).

We compared the procedure codes we identified to the procedure codes lists used in previous research. Young and colleagues (1) used 14 CPT codes to identify visits for psychosocial services in a study of treatment patterns for youth with psychiatric disorders enrolled in Mississippi Medicaid. Of the 14 procedure codes they listed, 12 were consistent with the list of codes we identified and were classified as assessment (*n* = 4), psychological testing (*n* = 1), group therapy (*n* = 1), and therapy (*n* = 6). One of their procedure codes (H2012) was not included in our outpatient services list as it refers to behavioral health day treatment. The other code (90618) was not found either in our dataset or in an online search. Winders Davis and colleagues (4) used 17 CPT codes to identify psychosocial therapy visits in their study describing patterns of service utilization among youth enrolled in Kentucky Medicaid and diagnosed with ADHD. Of these, 16 were included in our list and were classified as crisis therapy (*n* = 2), group therapy (*n* = 2), or therapy (*n* = 12). The remaining code, 90848, is a new procedure code for “prolonged services—psychotherapy” recommended for the CPT 2023 update by the AMA RVS Update Committee (RUC) in May 2021 (10). McGregor and colleagues (5) used 23 CPT codes to identify therapy services in their study describing racial and ethnic disparities in treatment for depression among adult Medicaid recipients in a nationally-representative sample. All were included in our list. We classified 5 of the codes as assessment, 6 codes as psychological testing, 2 codes as crisis therapy, 2 codes as group therapy, and the remainder as therapy (*n* = 8).

Overall, only 3 procedure codes identified in previous research were inconsistent with our list: 1 was not included in our list because it was not an outpatient service, 1 is a new code for 2023, and 1 was not found. In addition to classifying procedure codes identified in previous research according to service type, we identified and classified over 130 additional behavioral health service codes. Thus, previous research using a more limited set of procedure codes may not have characterized the full picture of behavioral health services received by youth.

Future researchers using billing data to examine behavioral health service utilization in their settings could use Table 1 to (1) identify procedure codes in their data set related to outpatient behavioral health and (2) classify their procedure codes by service type. If additional unidentified procedure codes are present, researchers could replicate our methods (i.e., review the AMA code descriptions and use our service classification definitions to classify the codes by service type) to fully capture the range of services provided to their population. Researchers interested in additional service types (e.g., psychiatric medication management, residential services) also could replicate our work by developing *a priori* service definitions and then reviewing the AMA code descriptions to identify and classify relevant procedure codes.

The current work should be examined in the context of some limitations. All procedure codes reviewed and classified were billed to Medicaid within one county. Billing practices may differ across geographic locations, and thus, it is possible there are behavioral health service codes that are used elsewhere but were not used in this county. We restricted the scope of the current study to outpatient behavioral health services, so the classification system may have limited utility for those seeking to examine utilization of more intensive services (e.g., inpatient, residential). Additionally, we did not include psychiatry or medication management services within our scope. Future work should similarly classify behavioral health medication management services to support research on the utilization of these services among youth. Despite these limitations, the current classification system expands upon methods used in previous research to identify behavioral health services by identifying and classifying 130 + additional behavioral health service codes. This system will support the work of health services researchers using administrative data to examine utilization of outpatient behavioral health services among youth. Further, using the service type language (e.g., assessment, outpatient therapy) in this system instead of complex procedure code language could facilitate understanding of administrative data for important stakeholders, such as patients and policymakers.

## Data Availability

Information for existing publicly accessible datasets is contained within the article. Further inquiries can be sent to the corresponding author.
